# *BIG LEAF* is a regulator of organ size and adventitious root formation in poplar

**DOI:** 10.1371/journal.pone.0180527

**Published:** 2017-07-07

**Authors:** Yordan S. Yordanov, Cathleen Ma, Elena Yordanova, Richard Meilan, Steven H. Strauss, Victor B. Busov

**Affiliations:** 1 School of Forest Resources and Environmental Science, Michigan Technological University, Houghton, Michigan, United States of America; 2 Department of Biological Sciences, Eastern Illinois University, Charleston, Illinois, United States of America; 3 Department of Forest Ecosystems and Society, Oregon State University, Corvallis, Oregon, United States of America; 4 Department of Forestry and Natural Resources, Purdue University, West Lafayette, Indiana, United States of America; Universidad Miguel Hernández de Elche, SPAIN

## Abstract

Here we report the discovery through activation tagging and subsequent characterization of the *BIG LEAF (BL)* gene from poplar. In poplar, *BL* regulates leaf size via positively affecting cell proliferation. Up and downregulation of the gene led to increased and decreased leaf size, respectively, and these phenotypes corresponded to increased and decreased cell numbers. *BL* function encompasses the early stages of leaf development as native BL expression was specific to the shoot apical meristem and leaf primordia and was absent from the later stages of leaf development and other organs. Consistently, *BL* downregulation reduced leaf size at the earliest stages of leaf development. Ectopic expression in mature leaves resulted in continued growth most probably via sustained cell proliferation and thus the increased leaf size. In contrast to the positive effect on leaf growth, ectopic *BL* expression in stems interfered with and significantly reduced stem thickening, suggesting that *BL* is a highly specific activator of growth. In addition, stem cuttings from *BL* overexpressing plants developed roots, whereas the wild type was difficult to root, demonstrating that *BL* is a positive regulator of adventitious rooting. Large transcriptomic changes in plants that overexpressed *BL* indicated that *BL* may have a broad integrative role, encompassing many genes linked to organ growth. We conclude that *BL* plays a fundamental role in control of leaf size and thus may be a useful tool for modifying plant biomass productivity and adventitious rooting.

## Introduction

In plants, final organ size is determined by the coordinated cell proliferation and expansion. Variation in leaf morphology due to environmental or genetic factors is highly correlated with leaf-cell numbers and, as a result, cell proliferation appears to be the main control point in the determination of final organ size [1]. However, manipulation of critical regulators of cell-cycle progression in mutant and transgenic plants has had little effect on organ size, primarily due to compensatory changes in cell expansion and/or differentiation [1, 2]. This suggests that regulatory mechanisms coordinating cell proliferation and growth determine final organ size. Genetic dissection in the model plant *Arabidopsis thaliana* and other herbaceous plants has revealed some insights into the mechanism and the factors involved. They include a variety of hormonal, metabolic, and regulatory cascades; transcription factors of various families, and their corresponding microRNAs; as well as signaling molecules with incompletely defined biochemical function [1, 2]. Final leaf size is influenced by several strictly regulated and coordinated processes, such as: number of cells in the primordia, rate of cell division, window of cell proliferation, and timing and rate of cell expansion [3].

A major regulator of organ size via regulation of cell proliferation in *A*. *thaliana* is the *AINTEGUMENTA* (*ANT*) gene [4–6]. Initially, *ANT* was shown to be involved in the regulation of flower development, but later work revealed its role in controlling the size of leaves and other organs (e.g., flower, siliques, roots, and seeds). *AINTEGUMENTA* is an AP2-domain transcription factor that belongs to a small subfamily of eight members, known as *ANT-like* (*AIL*), some of which have been functionally characterized [7]. The *Populus trichocarpa* ortholog of *AIL*, *PtaAIL1*, is involved in regulation of adventitious root development and bud phenology [8, 9]. *AINTEGUMENTA* is one of several genes, including *CURLY LEAF* (*CLF*), *APETALA 2* (*AP2*), *LEUNIG* (*LUG*), and *STERILE APETALA* (*SAP*), which negatively regulate expression of *AGAMOUS* (*AG*), in the first two whorls of developing flowers, as well as in vegetative organs [10–12]. Loss of function of these genes leads to small and/or curled leaves, with cells prematurely exiting the mitotic cycle [13–15]. In contrast to loss-of-function lesions, gain-of-function mutations in genes like *ANT* have the opposite effect: an increase in leaf size [4]. Recently, *SAP* was identified as F-box protein [16] involved in PEAPOD (PPD) protein degradation. PEAPOD1 and PEAPOD2 are negative regulators of leaf meristemoid cell proliferation in *A*. *thaliana* [17].

Here we show that a *Populus* ortholog of *SAP* influences leaf size, adventitious rooting, and the onset and extent of secondary growth.

## Materials and methods

### Plant material and statistical analyses

All experiments were performed using the *Populus tremula* x *P*. *alba* (genotype INRA 717-IB4). The plants were maintained *in vitro* on media as previously described [18]. For all analyses, three independent transgenic events (lines) were used. All growth parameters were taken on fully developed, healthy, greenhouse-grown plants. All statistical analyses were performed using Daniel’s XL Toolbox [19] for MS Excel (Microsoft). The number (n, individual plants measured from one growth experiment) of independent biological replicates for each analysis is given in the figures’ legends. In all cases the data are from three independent transgenic lines, except for the *blD* mutant and INRA 717-IB4 where data was collected for 3–5 plants per genotype. One-way analysis of variance was used to determine significance. Student’s t-test or Tukey’s multiple-comparison test was used to determine differences among the mean values.

#### Generation of activation tagging population and genomic positioning of the tag

Generation of an activation-tagging population was previously described [20]. Recovery of fragments flanking the insertion site and positioning of activation tag in the *Populus* genome (Phytozome.net) was performed as previously described [20].

### Binary vector generation and plant transformation

For recapitulation and production of *BL* over-expressing plants (*BL-oe*), the *BL* open reading frame (GenBank ID KR698934) was amplified using gene-specific primers with attached *Xho*I (5’) and *Xba*I (3’) restriction-site tails. The amplified fragment was cloned into the pCR4-TOPO vector using the TOPO-TA cloning kit (Invitrogen) and sequence-verified. The *BL* gene was inserted into the shuttle vector pART7 between the cauliflower mosaic virus (CaMV) 35S promoter (P35S) and the octopine synthase terminator (OCSt) using the *Xho*I-*Xba*I sites flanking the *BL* fragment. The P35S-*BL*-OCSt cassette was sub-cloned into the *Not*I site of the pART27 binary vector [21] before being transformed into *Agrobacterium tumefaciens* strain GV3101/pMK90 [22]. For the generation of the over-expression (oe) green fluorescence protein (GFP) and β-glucuronidase (GUS) fusion constructs, *BL-GFP*-oe and *BL-GUS*-oe, the *BL* open reading frame was amplified using gene-specific primers with attached attB Gateway sequence tail, as previously described [23] and cloned in binary vectors pMDC83 and pMDC140 [24], respectively. For generation of RNA interference (RNAi) lines (*BL*-i), the vector pK7GWIWG2(II) was used [25]. For generation of the *BL-SRDX*, a translational fusion with 12 amino acids of repressor domain SRDX [26] were incorporated in attB2 primer for the GATEWAY cloning (primer BL-R(-stop)DN) and cloned in binary vector pK7WG2 Karimi [25]. To produce lines containing *BL-GFP*-oe, *BL-GUS*-oe, *BL-SRDX*, and *BL-i*, *A*. *tumefaciens* strain AGL1 [27] was used for transformation. Generation of transgenic plants was performed as previously described [18, 20]. Transgenesis was verified via the polymerase chain reaction (PCR) for the presence of the transgene using the following primer pairs: p2735CI645/Fp2735CI645R (for *BL-oe*), GFPf/GFPr (for *BL-GFP*-oe), GUSf/GUSr (for *BL-GUS*-oe), and NPTf/NPTr (for *BL*-I and *BL-SRDX*). The sequences of all primers used are shown in S1 Table.

### RNA extraction and gene expression analyses

Extraction of total RNA, cDNA synthesis, and transcript quantification were performed as previously described [23, 28]. Quantitative RT-PCR was performed using a StepOnePlus Real-Time PCR System (Thermo Fisher Scientific) and the primers shown in S1 Table. Reaction mixtures contained Maxima SYBR Green qPCR master mix (Thermo Fisher Scientific), 0.2 μM of each primer, and 2 μL 10× diluted cDNA in a final volume of 20 μL. The default StepOnePlus cycling parameters were used. RT-PCR gel images were obtained using a GelDoc-It (UVP) documentation system and expressions were quantified using ImageJ (http://rsb.info.nih.gov/ij), as reported previously [23]. Three biological replicates were analyzed for each sample, and relative transcript abundance (expression) was calculated using ubiquitin as an internal standard [23, 29, 30].

### Microscopy and in-situ localization

For *in situ* RT-PCR, primers BLrt-f and BLrt-r were used (S1 Table). Cell area of adaxial epidermal cells were measured as previously described [31]. Shortly, mature leaves impressions were prepared with clear nail polish close to the middle of leaf near to the midrib, and from adaxial leaf surface. All impressions were fixed on glass slides, and examined under a phase contrast light microscope [31]. Tissues preparation, microscopy and *in-situ* localization were performed as previously described [28]. A confocal microscopy system, consisting of Spinning Disk Confocal Head (Yokogawa CSUX1), a Nikon Eclipse Ti inverted microscope outfitted with 10x and 60x oil immersion lenses, and a precision motorized stage, was used for sub-cellular localization of GFP in leaf cells from stably transformed *BL-GFP*-oe poplar. The system is coupled with a high-resolution, high-frame-rate camera (Photometrics Cool SNAPHQ2). Laser light sources include wavelength lines of 488, 561, 642 nm. The system was controlled with Metamorph software.

### Sequence and phylogenetic analyses

Sequence homology searches and analyses were performed using the Phytozome (http://www.phytozome.net/poplar.php) and the National Center for Biotechnology Information BLAST server (http://www.ncbi.nlm.nih.gov/BLAST/). Protein sequence was downloaded locally, aligned by ClustalW method [32], and analyzed all using MEGA4 [33]. For construction of phylogenetic tree, the genetic distance method with the neighbor-joining approach was used. Confidence estimates for each branch of the resulting trees were statistically tested by bootstrap analyses of 1,000 replications, using MEGA4 software.

### Microarray analyses

Native expression of *BL* occurs in apical tissues, and observed phenotypic differences were confined mostly to leaves and stems. Thus, we collect two bulk samples (six plants/bulk, two plants from each of the three *BL-oe* lines,) from apices, stem (30^th^ internode), and leaves (30^th^ node). Collected tissues were flash-frozen in liquid nitrogen and stored at -80°C. Extraction of total RNAs were performed as previously described [28]. Microarray data were collected and analyzed according to MIAME standards [34] and deposited at GEO (GSE68859). The labeling, hybridization, and imaging procedures were performed according to Affymetrix protocols at the Center for Genomics Research and Biocomputing at Oregon State University (http://corelabs.cgrb.oregonstate.edu/affymetrix), using the Affymetrix Poplar GeneChip as previously described [23, 28]. Raw data were normalized using the RMA algorithm [35] and further analyzed statistically using TM4:MeV software [36, 37], utilizing Affymetrix probe annotation described in [38]. To identify differentially regulated genes/probes (DEG), we implemented LIMA analysis [39] with significance at False Discovery Rate FDR <0.05 between *BL-oe* and wild-type INRA 717-1B4 (WT-717). Gene Ontology (GO) analyses were done using the corresponding *A*. *thaliana* gene ID in AgriGO [40], with FDR<0.05.

## Results

### Isolation of a poplar activation tagged mutant with increased leaf size

**Fig 1:**
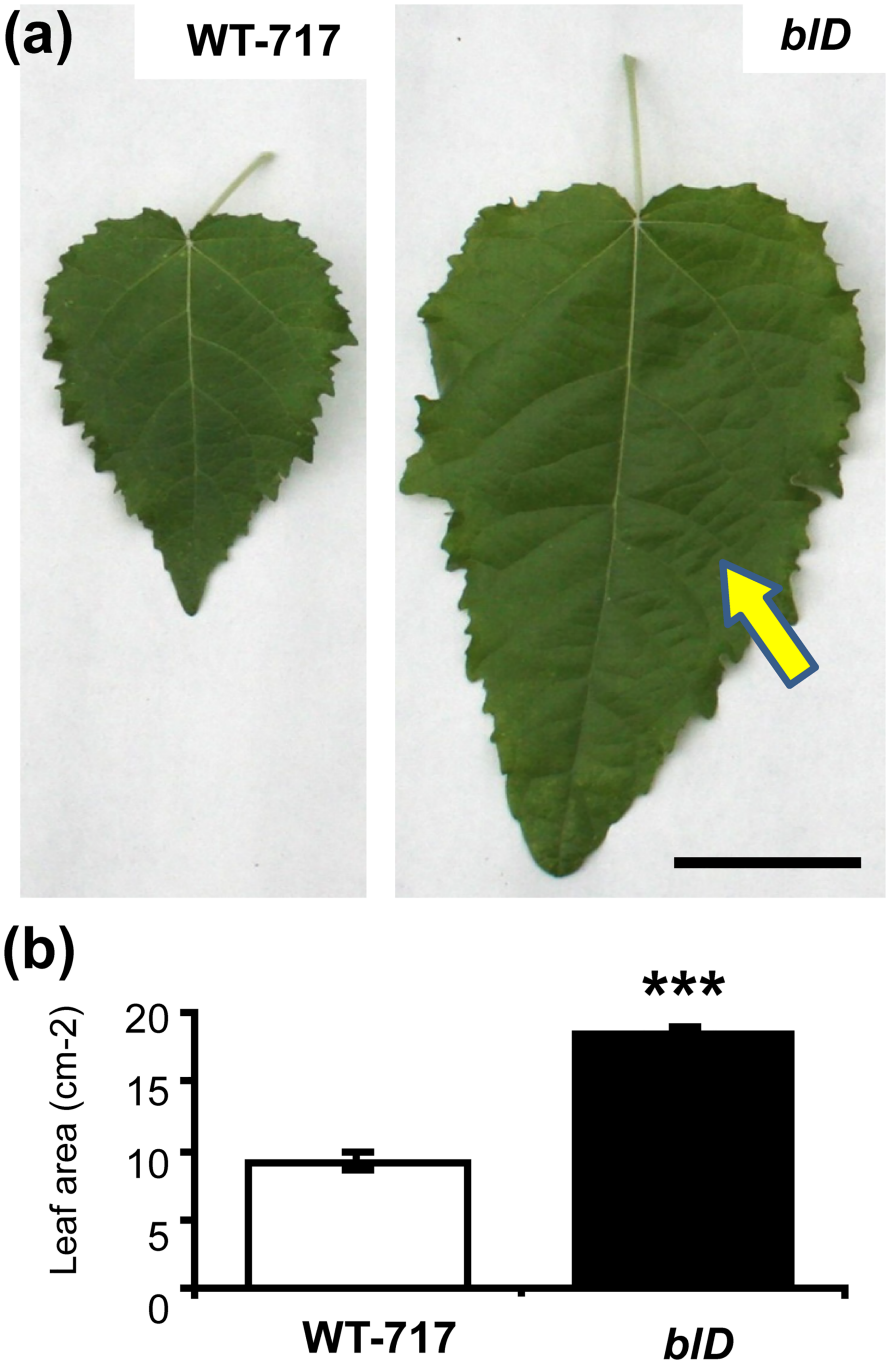
The *blD* activation tagging line with increased leaf size. (a) Leaves from *blD* mutant line plants in the greenhouse show increased leaf size, when compared to wild-type plants (WT-717). Arrow, pinpoint to area with uneven surface of the leaf lamina. (b) Leaf area of greenhouse-grown plants from *blD* and WT-717 (bars represent standard error (SE), n = 5, ***—Student t-test P <0.001). Scale in (a) and (b) = 5 cm.

A mutant with increased leaf size was identified in a population of 627 activation-tagged poplar events [20, 41] (Fig 1). The *big leaf dominant* (*blD*) mutant was named for its predominant phenotype. In addition to increase in leaf size (Fig 1b), we also observed an uneven leaf surface (Fig 1a), a phenotype often associated with lesions in the coordination of cell proliferation and organ growth [42, 43]. Measurements after two full growing seasons in the field showed no change in height, but a statistically significant decrease in stem diameter compared to wild-type 717-1B4 (WT-717) (S1 Fig). Scanning electron microscopy and measurements of the epidermal cell area revealed no significant difference in cell size on the abaxial part of the leaves from two mutant and WT plants (S1 Fig). Therefore, we concluded that the observed phenotype is likely a result of increased cell proliferation, not cell size.

### Identification and molecular characterization of the activation tagged gene

**Fig 2:**
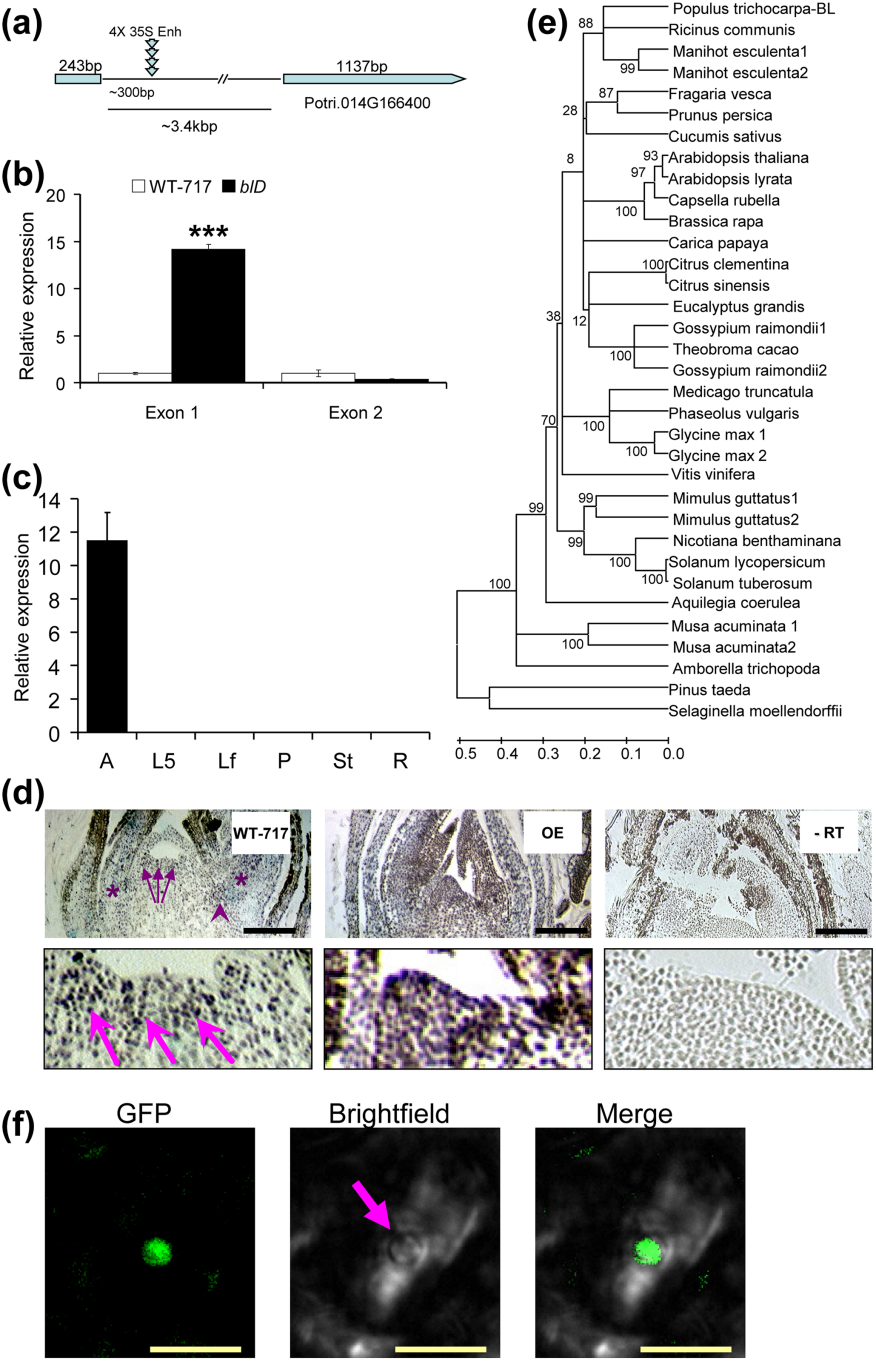
Molecular characterization of the *blD* tagged gene. (a) Schematic representation of the activation-tagging insertion in the *Populus* genome. (b) Expression verification of *BIG LEAF* (*BL*) activation. (Bars represent means ±SE, n = 5, ***—t-test p<0.001). (c) *BL* is expressed only in the apical part of the poplar tree. Tissues were collected from WT-717 plants at the same time of the day and correspond to: 1 cm of the root tips (R); 5 mm of apical shoot, including the meristem and subtending leaf primordia (A); incompletely expanded young (Leaf plastochron index [45], LPI 5) leaves (L5); fully expanded, mature (LPI 10) leaves (Lf); petioles of fully expanded leaves (P); whole stem collected from internodes of LPI 15–20 (St). In (b) and (c) relative expression was determined via qRT-PCR using ubiquitin as a loading control. Bars show means ± SE (n = 3). (d) *In situ* RT-PCR localization of the *BL* transcript in apices of WT-717 (left), *BL* over-expressing plants (middle). WT-717 apices served as negative controls (right). In WT-717, arrows indicate the localization of the *BL* transcript in the meristem and leaf primordia; arrowhead and star indicate localization in axillary meristem and newly formed leaves, respectively. Lower panels show magnified meristem areas. (e) Phylogenetic analysis of BIG LEAF/STERILE APETALA proteins. The tree was generated using Neighbor-Joining method with bootstrap confidence based on 1,000 iterations. (f) Nuclear sub-cellular localization of BL-GFP in leaf cell from a stably transformed *BL-GFP*-oe transgenic poplar. Pictures represent epi-fluorescence (GFP), black-white field (bright field), and a merged image. Scale bars in (d) = 200 μm, (f) = 25 μm.

We recovered sequence flanking the left border of the transfer DNA (T-DNA) insert (GenBank ID KR698933). Repeated attempts to identify an alternative insertion site using various TAIL-PCR primers and plasmid rescue always detected the same position, suggesting that this is a single insertion event. BLASTn searches mapped the insertion to the Potri.014G166400 gene model on chromosome 14 (Fig 2a). T-DNA was inserted in the intron 254 base pairs (bp) downstream of the border with the first exon. Exon 1 was highly up-regulated in the mutant plant (Fig 2b); however, exon 2 showed approximately equal expression in mutant and WT-717 plants (Fig 2b). It appears that enhancers in the flanking intron sequence activated the promoter of the corresponding gene, and that transcription likely ceased prematurely in one of the multiple terminator sequences of the T-DNA inserted in the intron. Despite numerous attempts, we were unable to clone the aberrant transcript using 3' RACE with gene-specific primers targeting exon 1. Homology searches with the predicted protein sequence showed that the putative *BL* gene encodes a protein with high similarity (58% amino acid identity) to *A*. *thaliana SAP* (AT5G35770), which is involved in inflorescence, flower, and ovule development [12, 44]. In both *A*. *thaliana* and poplar, *SAP* is encoded by single genes. *BIG LEAF*/*SAP* homologues are uniquely present in all vascular plants, including *Selaginella*. The *BL*/*SAP* gene is not present in any *Poaceae* spp. with sequenced genomes, or in any grass ESTs. However, *BL*/*SAP*-like genes were found in monocotyledonous banana (*Musa acuminata*) (Fig 2e), and *Cycadophyta* species *Zamia vazquezii* (GenBank FD768395.1, FD768570.1; partial sequence not included in analyses). In poplar, *BL* is expressed almost exclusively in apical tissues (Fig 2c). *In situ* localization of *BL* transcripts pinpointed its localization to the apical meristem and newly formed leaf primordia, as well in the axillary meristem and very young leaves (Fig 2d).

### Transgenic modifications recapitulate the BL phenotype

**Fig 3:**
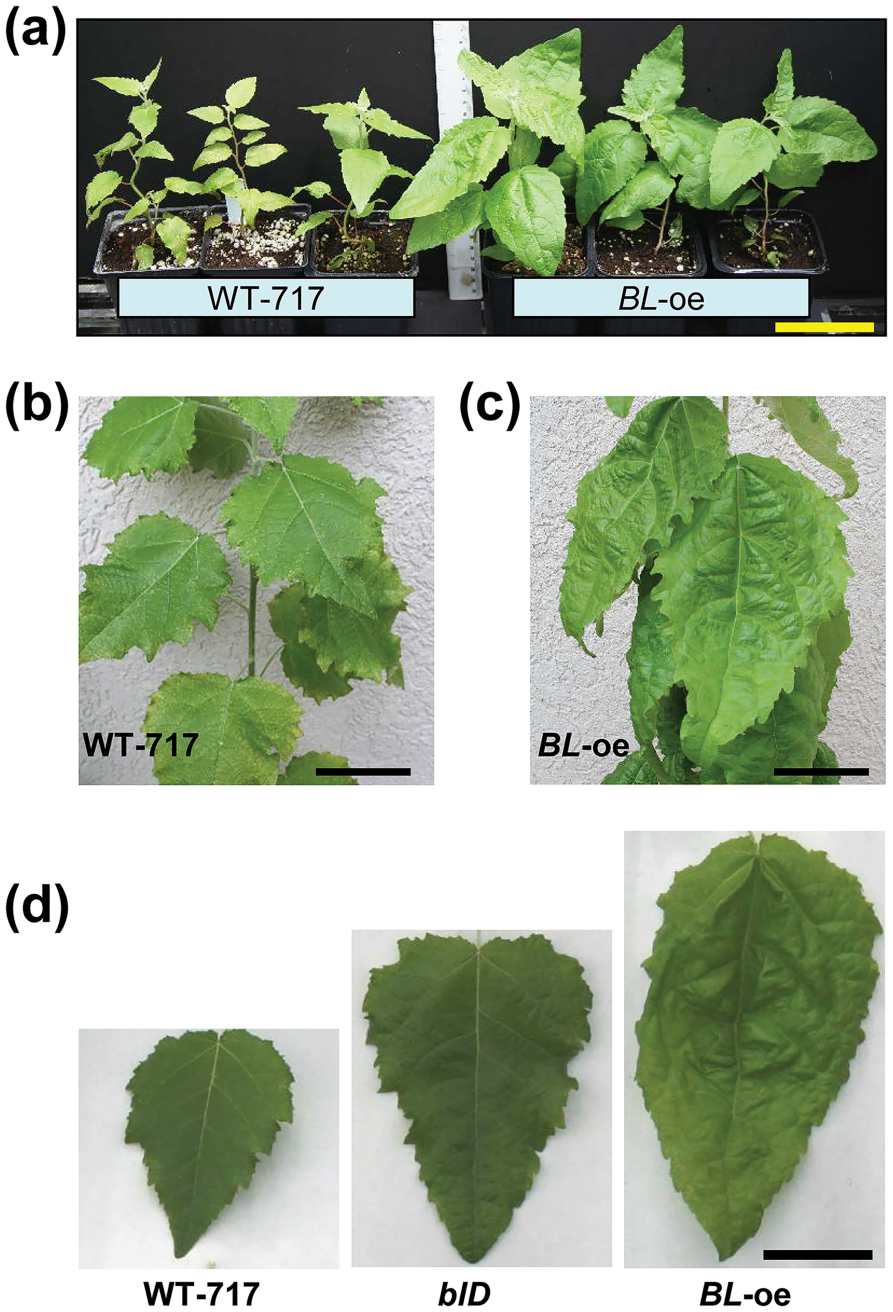
Recapitulation of the mutant *blD* phenotype. (a) One-month-old plants, in the greenhouse, exhibited increased leaf size in the recapitulation lines (*BL-oe*). (b) and (c) Close view of leaves at the 15-20^th^ internodes in six-month-old plants display visibly increased leaf size in *BL-oe* (c) compared to WT-717 (b). (d) Representative leaves (at the 20^th^ internode) from WT-717, *blD*, and *BL-oe* plants. Scale bars (a)-(d) = 5 cm.

**Fig 4:**
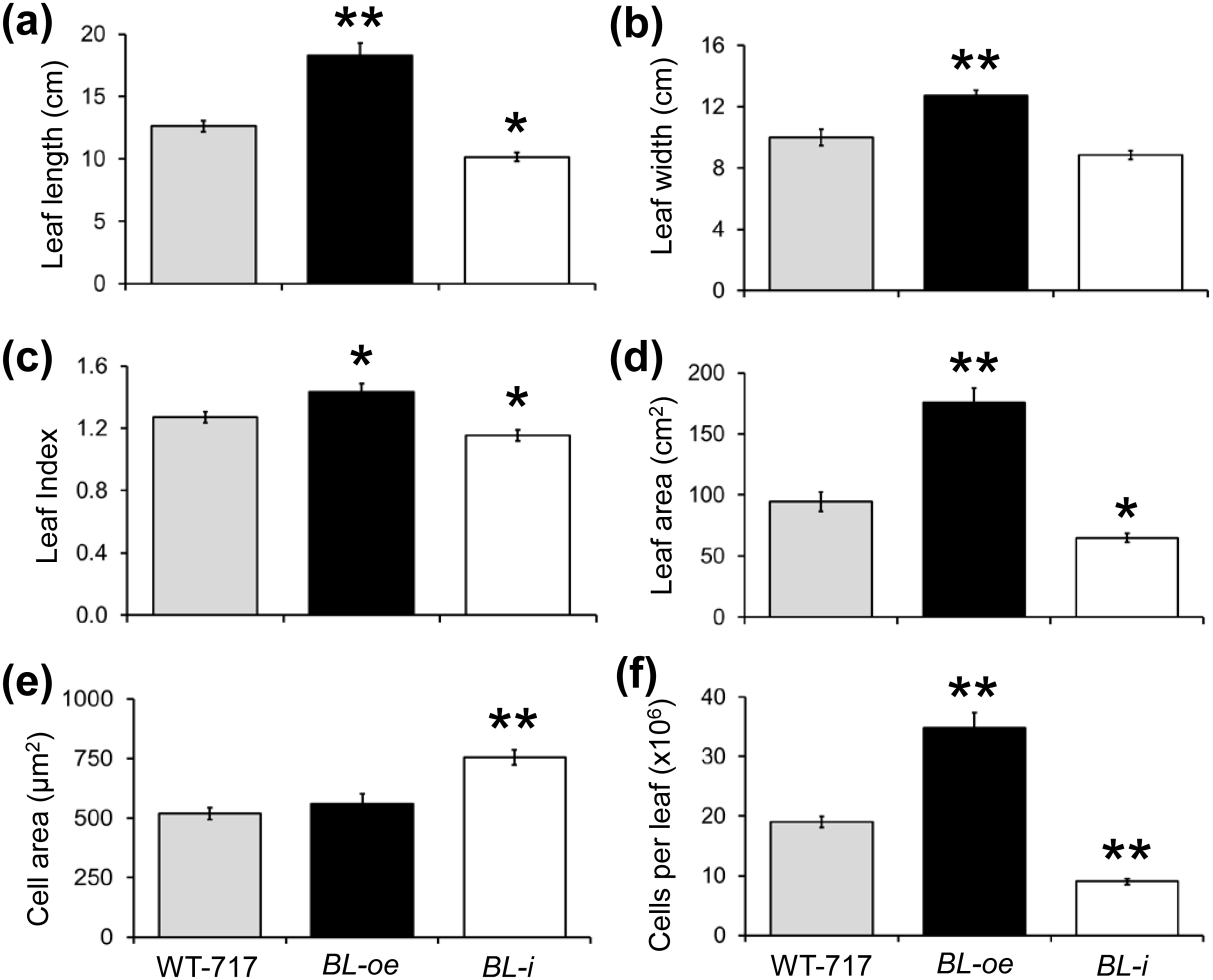
Characterization of leaf size and shape in *BL*-over-expressing (*BL-oe*) and down-regulated (*BL*-i) plants. Leaf parameters were measured on fully developed leaves subtending the 15^th^ to 20^th^ internodes from greenhouse-grown plants. (a) Leaf length. (b) Leaf width. (c) Leaf Index calculated from (a) and (b). (d) Leaf area. (e) Cell area of adaxial epidermal cells. (f) Total cells per leaf calculated from (d) and (e). Error bars represent mean ±SE (n = 5, in (e) n = 20). Asterisks indicate significance as determined by Student’s t-test, with * and ** denoting P < 0.05 and P < 0.01, respectively.

Despite the unusual activation by insertion into intron, because of the significance of the phenotype, we attempted recapitulation by transforming full-length cDNA of the *BL* coding region into WT-717 (S2a Fig). We regenerated more than 40 lines with the over-expression construct (*BL-oe*). Across all events, we observed leaf phenotypes ranging from those seen in the original *blD* mutant to larger increases in leaf size and more severe alterations (uneven leaf surface) in leaf lamina (Fig 3). *BIG LEAF* over-expressing plants (S2b Fig) displayed significant increases in several leaf size characteristics, including: length, width, length/width ratio, and area (Fig 4a–4d). We also produced RNAi lines (*BL*-i) to suppress expression of *BL* (S3 Fig). For all lines in which *BL* expression was down-regulated, we observed small but significant reductions in leaf length (Fig 4a), leaf area (Fig 4d), and length/width ratio (Fig 4c).

### BL transgenic manipulations affect cell proliferation

To better understand the changes in leaf morphology, we measured cell number and size in the three genotypes (WT-717, *BL-oe*, and *BL*-i). *BIG LEAF* over-expression led to a significant increase in cell number, whereas down-regulation had the opposite effect (Fig 4f). In addition, cell area was significantly increased in *BL*-i plants (Fig 4e), suggesting a compensatory effect [46]. Moreover, *BL* overexpression caused shoots regeneration from callus tissues that typically does not produce shoots (S2c Fig). Thus, the *BL* effect on poplar leaf size is largely mediated through regulation of cell proliferation.

### BL expression affects leaf growth and differentiation

**Fig 5:**
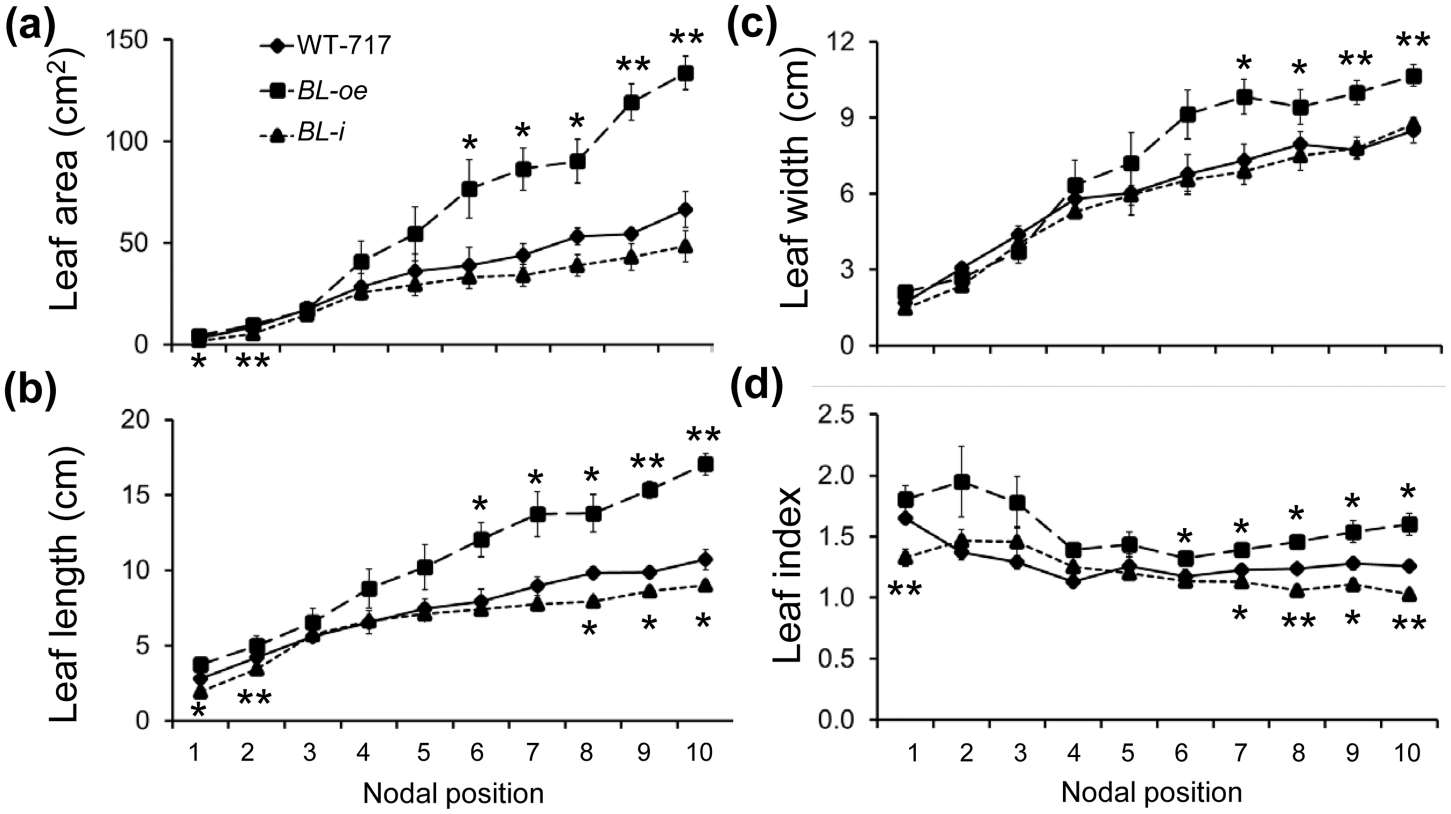
Dynamics of leaf growth in *BL*-over-expressing (*BL-oe*) and down-regulated (*BL*-i) plants. Leaf parameters were measured from the 1^st^ to the 10^th^ fully developed leaves. (a) Leaf area. (b) Leaf length. (c) Leaf width. (d) Leaf Index calculated from (b) and (c). Bars show mean ±SE (n = 3), * and ** represent statistical differences determined by Student’s t-test at p <0.05 or 0.01, respectively. Asterisks at the bottom indicate significance of the *BL*-i and at the top of *BL-oe* when compared with WT-717.

**Fig 6:**
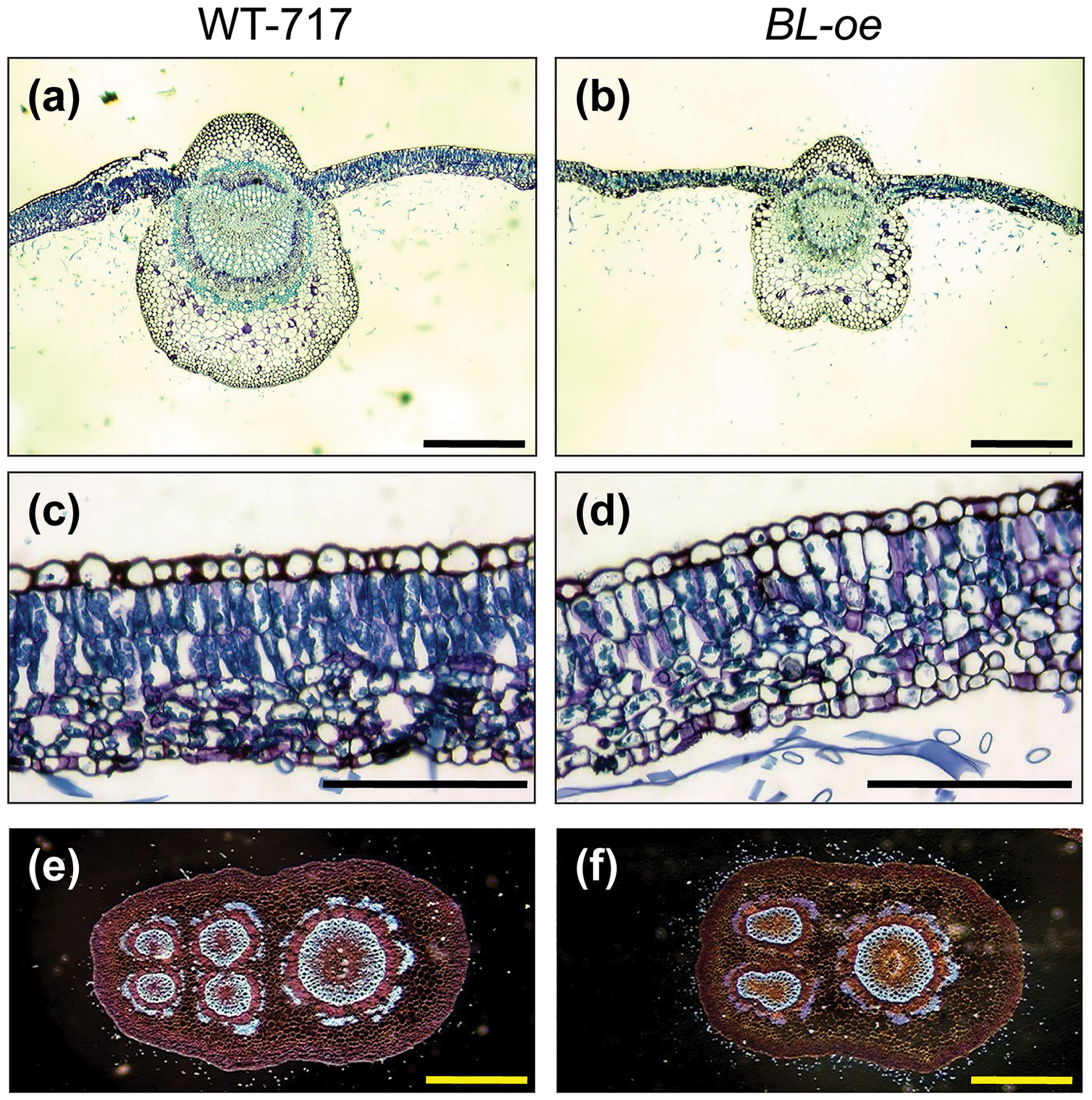
Anatomical changes in the leaf blade and petiole of *BL-oe* plants. The panels show leaf sections from WT-717 (left) and *BL-oe* (right) plants. All sections were stained with toluidine blue. (a) and (b) show the leaf midvein. Note the smaller midvein and reduced lignification (intensity of the blue staining) in *BL-oe*. (c) and (d) show the cross-section of leaf lamina. (e) and (f) show cross-sections of petioles. Images were obtained for better clarity under phase contrast. Scale bar in (a), (b), (e) and (f) = 500 μm; in (c) and (d) = 100 μm.

We investigated the dynamics of leaf growth by taking measurements at different nodal positions, marking the developmental transition from young, actively growing (upper-most nodes) to mature, fully differentiated leaves that have achieved their final size. As early as the first two internodes, significant differences were observed in the leaf length (31% at the first, 18% at the second internode) and area (40% at the first, 35% at the second internode) of *BL*-i plants (Fig 5a and 5b). This is consistent with the native expression of *BL* in the apex, localized to the very young leaves and nodes (Fig 2c). In contrast, significant changes in leaf size of *BL-oe* plants were found further down the developmental gradient, starting around internode 6 (Fig 6a–6d). In addition, the leaves of *BL-oe* plants were lighter green and the leaf lamina were thinner (36.3±3.3% reduction, p<0.001, S2 Table) than WT-717 (Fig 6a and 6b). *BL-oe* leaves also had poorly differentiated, unorganized mesophyll layers. Consistent with the lighter green color, the cells from the mesophyll palisade in *BL-oe* transgenics contained visibly fewer chloroplasts per cell (61.4±2.8% reduction, p<0.001, S2 Table), with some cells almost completely devoid of chloroplasts (Fig 6c and 6d). The xylem in the mid-vein and petiole of *BL-oe* plants was less lignified as evidenced by the lighter toluidine blue staining (Fig 6b) and fainter fluorescence under phase contrast (Fig 6f) [47, 48]. The petioles of WT-717 plants have four amphicribral bundles near the main vein (Fig 6e), while *BL-oe* plants have only two (Fig 6f), demonstration additional changes in vasculature development. In summary, leaf differentiation was disrupted in response to *BL* up-regulation.

### BL translational fusions and protein localization

Sequence analyses suggested that *A*. *thaliana SAP* might be a transcriptional regulator [12] and was recently identified as an F-box protein involved in PPD protein degradation [16]. Based on these results, also we produced transgenic lines expressing a translational fusion of *BL* with *GFP* (S4a Fig, *BL-GFP*-oe,), and found BL localized to the nucleus (Fig 2f). To further characterize BL protein, we produced multiple lines in which a fusion of GUS and the SRDX repressor domain [26] was over-expressed. Interestingly, all *BL-SRDX*-oe lines developed leaves comparable to WT-717 (S5 Fig), indicating that the SRDX fusion completely suppresses the positive effect of BL on leaf growth. In contrast, translational fusions in *BL-GFP*-oe (additional 165 amino acids) and *BL-GUS*-oe (additional 603 amino acids) transgenics displayed significantly increased leaf size, similar to the *BL-oe* lines (S4a Fig), indicating that the additional amino acids do not interfere with BL functionality, as does the SRDX repressor domain. SRDX is only a 12 amino acids repressor domain motive and when present can convert transcriptional activator to repressors (a dominant negative version), and also can affects the protein-protein interaction [26].

### BL interferes with stem growth and development

One striking characteristic of both the *blD* mutant and *BL-oe* plants was a significant reduction in stem diameter (S1 and S6 Figs). We, therefore, studied stem anatomy at several internodes of WT-717, *blD*, *BL-oe*, and *BL*-i plants (S6 Fig). The internodes were chosen to represent the developmental and growth transition from primary (elongation), at internode 5, to secondary (lateral thickening), at internodes 10 and 20, growth [23, 49, 50]. The main differences were observed in xylem development (S6k Fig). In contrast to WT-717, xylem growth and differentiation of *blD* and *BL-oe* plants was severely curtailed across all of the analyzed internodes. In addition, phloem fiber lignification was also significantly reduced as evidenced by lack of fluorescence under phase contrast (S6B Fig). We also observed increased internodes number (S6l Fig) and reduced height (Fig 6m) in *BL-oe* transgenic lines. Down-regulation of *BL* (in *BL*-i plants) had no significant effect on secondary growth and development (S6 Fig).

### BL positively affects adventitious rooting

**Fig 7:**
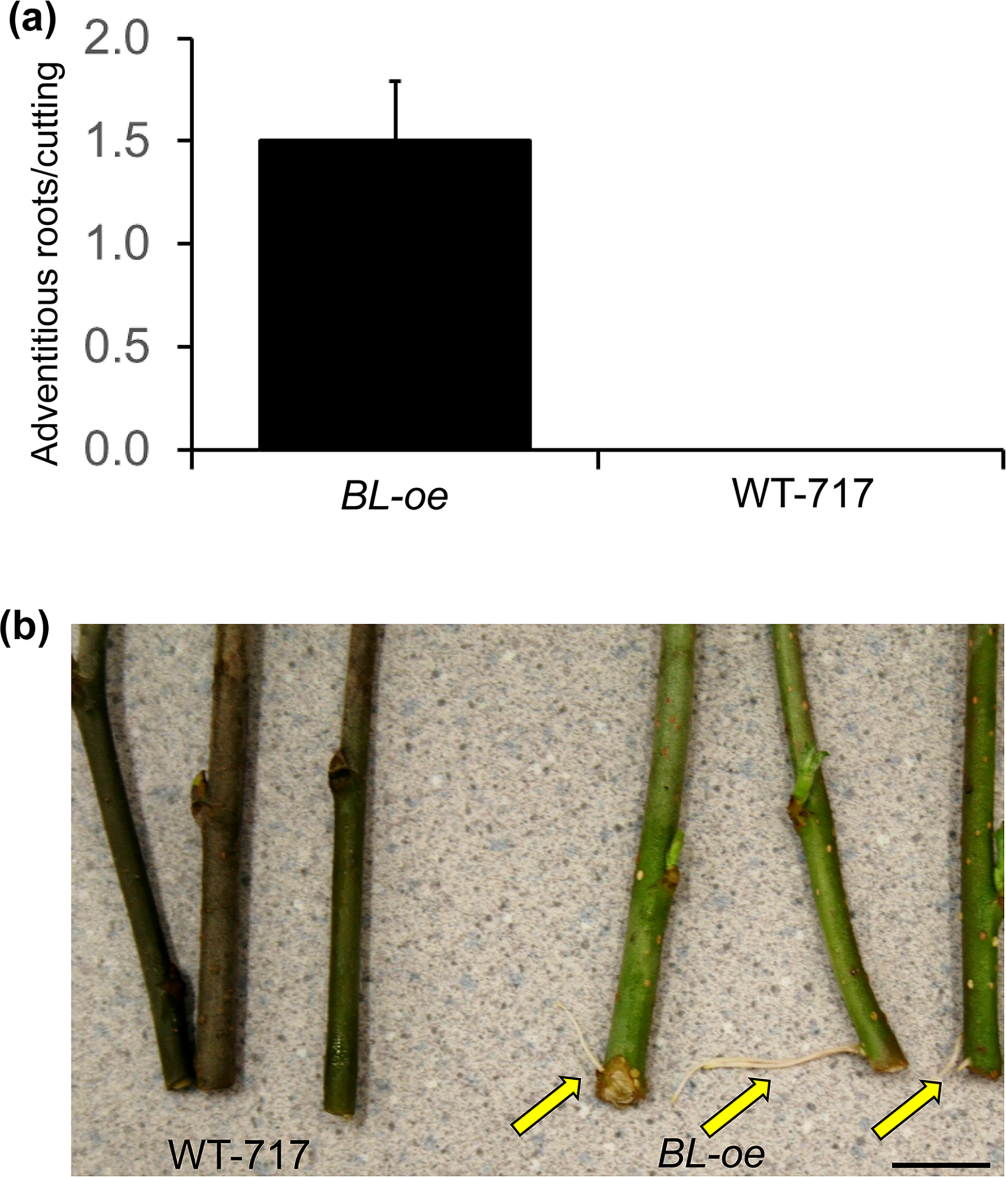
*BIG LEAF* positively affects adventitious rooting. (a) *BIG LEAF* over-expression increases adventitious rooting of stem cuttings. Bars show means ± SE (n = 3). (b) Two-weeks-old cuttings show adventitious rooting in *BL-oe* but not WT-717 plants. Scale bars is 1 cm.

We investigated the rooting capacity of *BL-oe* plants by placing dormant cuttings in water for one month. The transgenics exhibited rooting capacity at the second week (Fig 7b) while WT-717 stem material did not formed roots for a month (Fig 7a). Observed changes in *BL* transgenics in growth, cell number, and size resemble changes observed in *A*. *thaliana ANT* mutants and transgenics [4]. Furthermore, the poplar ortholog of *ANT* (*PtAIL1*) was found to be a positive regulator of adventitious rooting [8]. This prompted us to study the expression of *PtAIL1* in the transgenic plants (S7 Fig). Indeed, *PtAIL1* expression was significantly up- and down-regulated in *BL-oe* and *BL*-i plants, respectively (S7 Fig). Therefore, the observed *BL* growth-promoting and adventitious root-inducing effects might be mediated via modulation of *PtAIL1* expression.

### Transcriptome changes underlying the BL phenotype

For a more complete understanding of the *blD* phenotype, we employed genome-wide microarray analyses. We compared the transcriptomes of *BL-oe* and WT-717 plants in three types of tissues: the shoot apex, mature leaves, and stems (30^th^ internode). These analyses identified a total of 8,313 differentially expressed genes (DEGs) (LIMA test, FDR<0.05), from which 2,494 (1023 up-, and 1471 down-regulated) DEG were in the apex, 4,782 (2595 up-, and 2187 down-regulated) were in the leaves, and 2,354 (786 up-, and 1568 down-regulated) were in the stems (S1 Data). Microarray results were validated for nine genes (*BIG LEAF plus 4 up- and 4 down-regulated*, *randomly chosen genes*) using qRT-PCR (S8 Fig). Only 124 DEG were common to all three tissues (S2 Data). The GO analyses of all DEG in the different tissues (S3 Data) indicated common enrichment categories (S4 Data) for genes involved in response to stimulus, metabolic, and cellular process; biological regulation; and development (S4 Data). Among GO categories related to plant development were genes involved in meristem, leaf, and flower development and function (S4 Data).

Genes involved in regulation of organ growth [51, 52] were significantly affected by the *BL* over-expression (S5 Data). Genes which are positive regulators of organ growth, such as *KLU* (*KLUH*), *ANT*, *TARGET OF RAPAMYCIN* (*TOR*), *ANGUSITFOLIA3* (*AN3*)-*like*, and *GROWTH REGULATING FACTOR* (*GRF*)-*like*, were significantly up-regulated in the leaf tissues (S3 Data). SAP has been shown to regulate PPD at the protein level via 26S proteasome degradation [16]. Surprisingly, poplar *PPD* homologs (Potri.005G214300: PtpAffx.37038.1.A1_at, Potri.002G048500: PtpAffx.201708.1.S1_at) were significantly up-regulated at the transcriptional level in *BL-oe* leaves (S1 Data).

Among all DEGs, we identified genes which are positive regulators of root growth that were specifically upregulated in the stems of *BL-oe* plants (S6 Data), including: *MONOPTEROS/auxin response factor 5*, *ARF17*, and *NAC1* [53–55].

## Discussion

Here we report the discovery and characterization of *BIG LEAF* (*BL*), a novel regulator of leaf size in poplar, named after the dominant, gain-of-function phenotype identified in an activation-tagged line. *BL* is an ortholog of the *A*. *thaliana SAP* gene [12, 16, 44]. *BL*/*SAP* is a plant-specific gene whose lineage can be traced as far back as the lycophyte *Selaginella moellendorffii*; in most species with sequenced genomes, there are usually one to two copies (Fig 2d). A homologous gene has not been found in any of the *Poaceae* species that have been sequenced, but it is present in other monocots, such as banana and date palm (Fig 2e). In *A*. *thaliana*, *SAP* was first shown to be essential for flower development and megasporogenesis [12]; however, the presence of *BL*/*SAP* paralogs in non-flowering *S*. *moellendorffii* is an indication that it may be involved in developmental processes other than flower development. In *A*. *thaliana* SAP was identified as a F-box protein that regulates organ size via degradation of PEAPOD proteins [16]; PEAPOD proteins are absent in *Poaceae* species [17, 56]. It has been proposed that the *SAP* and *PPD* genes evolved to regulate development of merisemoid cells in dicots, and modifying *SAP* expression in *A*. *thaliana* revealed that it positively affects leaf size [16], and flower size in *Capsella* [44].

In the present study, we show that over-expression of *BL* in poplar leads to greatly increased leaf size, while down-regulating *BL* leads to a slight but significant reduction in leaf size. The only slight reduction in leaf size is likely a result of an increase in cell size (Fig 4e). Such compensatory effects have been observed in leaves of irradiated plants, or in plants in which the growth-promoting genes, such as *AN3* and *ANT*, were mutated [57], but was not seen in *A*. *thaliana SAP* loss-of-function mutant [16]. However, recently identified new loss-of-function alleles of SAP [16] led to same reduction of leave size as described here plants with down-regulated *BL* (*BL-i*, Fig 4).

Involvement of *BL* in the regulation of leaf size in poplar is also supported by the increased expression of major regulators of leaf growth in the *BL-oe* plants (S6 Fig, S5 Data). For example, the poplar ortholog of *ANT*, *PtaAIL1*, a major positive regulator of organ growth [51], was up-regulated in *BL-oe* and down-regulated in *BL*-i plants (S4 Fig, S5 Data). The changes in *PtaAIL1* expression are even more significant given that *SAP* is a part of a complex mechanism that regulates *AG* expression in *A*. *thaliana* [10, 11]. This suggests that ANT and BL/SAP may be part of a common module involved regulating leaf size through a yet uncharacterized mechanism involving protein degradation, given that SAP is an F-box protein [16]. The role of BL in determining leaf size in poplar involves regulation of the very early stages of leaf outgrowth, around or during leaf primordia initiation and outgrowth. This notion is reinforced by the localization of gene expression in the SAM, particularly leaf primordia, and its almost complete absence from the later stages of leaf development (Fig 1). Furthermore, and more importantly, *BL* knock-down via RNAi resulted in a reduction in leaf size during the very early stages of leaf outgrowth (Fig 5). In contrast, ectopic expression of *BL* in mature leaves, where *BL* is usually not expressed, resulted in continuous growth and, thus, increased leaf size (Fig 5). Leaf size is determined by cell proliferation and cell growth. *BIG LEAF* function clearly involves regulation of cell proliferation (Fig 4 and S2c Fig). *BIG LEAF* over-expression in poplar led to increased cell number, while down-regulation had the opposite affect (Fig 4e). In summary, *BL* regulates leaf size in poplar by positively regulating cell proliferation during the very early stages of leaf outgrowth, similarly to effect of *SAP* in *A*. *thaliana* [16].

In addition to leaf size, we showed that *BL* has a strong, positive effect on adventitious root formation. Adventitious rooting is important for vegetative propagation in many crops and can greatly affect the deployment of clonal material [8, 58, 59]. We show that *BL* expression is correlated with the transcript abundance of *PtAIL1*, which was previously shown to regulate adventitious root formation [8], further implicating *BL* in the regulation of this process in poplar.

In sharp contrast to the positive effect on cell proliferation and organ growth in leaves and adventitious roots, *BL* appears to have a strong inhibitory effect on stem diameter, specifically xylem formation (S6 Fig). Given that native *BL* expression is not detected in stems, this effect seems to be the result of the ectopic expression of *BL*. This outcome shows the highly specific nature of organ-size regulation; a positive regulator of leaf growth can act as a repressor in the context of stem thickening. Given that *BL* inhibited the development of leaf veins and petioles (Fig 6), the suppression of xylem growth may be the result of *BL* delaying or reducing vascular differentiation.

BIG LEAF and SAP protein sequences contain domains that are typically found in transcriptional regulators [12] and the latter was recently identified as F-box protein [16]. Using a *BL-GFP* fusion protein, we showed that BL protein is localized in the nucleus (Fig 2e), same is true for *A*. *thaliana SAP* [16]. Furthermore, we showed that fusion of BL with the strong SRDX repressor domain rendered the protein non-functional, as evidenced by the wild-type-like phenotype of the *BL*-SRDX over-expressing transgenics (S5 Fig). In sharp contrast, fusion of BL with either the GFP or GUS proteins led to the same phenotype as *BL*-over-expressing transgenics (S4 Fig). This suggests that SRDX has specific inhibitory effect on BL function by interfering with putative protein-protein interactions [60], and can be utilized to study BL protein-protein interactions.

The strong promoting effect of BL/SAP on leaf growth is of interest in relation to increased productivity in lignocellulosic bioenergy and other types of crops. However, the negative effects associated with the strong ectopic expression needs to be addressed before the gene can be used as a tool to enhance productivity. This can be achieved, for example, by using tissue/organ-specific expression and/or more moderate up-regulation of the gene.

## Supporting information

S1 Table Primers used in this work. (PDF)

S2 Table Leaf thickness and chloroplast number/cell in *BL-oe*. (PDF)

S1 Fig Biometrical characterization of *blD* mutant under field conditions. Graphs present data for the plants height (top), stem diameter (middle), and leaves adaxial epidermis cell area (bottom) are shown. Error bars represent mean ±SE (n = 4, n = 25 for cell area), asterisk indicate significance P<0.05 as determined by Student’s t-test.
(TIF)

S2 Fig Expression and proliferation of *BL-oe*. (a) Schematic representation of the construct used for transformation. Backbone plasmid is pART27. P35S = 35S promoter from the Cauliflower Mosaic Virus, ocsT = octopine synthase terminator, KmR = gene cassette for kanamycin resistance in plants, R and L are right- and left-hand T-DNA borders. (b) RT-PCR expression analyses of *BL* in apical shoots from six, randomly chosen transgenic *BL-oe* lines (1 to 6) reveal strong up-regulation of the gene in all tested lines. *Ubiquitin* (*UBI*) was used as a loading control. (c) Spontaneous shoot outgrowth from cambium-derived callus in *BL-oe* transgenic plants (left panel), observed in about 15% (with 1–4 shoots) of the plants. WT-717 (right panel) formed regular callus to seal the wound but no shoot outgrowth was observed.
(TIF)

S3 Fig RNAi *BL* knock down. In upper panel is shown representative RT-PCR demonstrating down-regulation of *BL* expression in apex from three transgenic lines. *Ubiquitin* (*UBI*) is used as a loading control.
(TIF)

S4 Fig Leaf phenotype of *BL-GUS*-oe and *BL-GFP*-oe transgenic plants. Over-expression of BL fused to either GFP (a) or GUS (b) fully recapitulated the *BL-oe* phenotype. Leaves shown are from three independent transgenic lines. Scale bar = 10 cm.
(TIF)

S5 Fig Leaf characterization in *BL-SRDX*-oe plants. Leaf parameters were measured from 15^th^ to 20^th^ fully developed leaf. (a) Leaf length. (b) Leaf width measured at leaf center. (a) Leaf Index calculated from (a) and (b). (d) Leaf area. (e) Cell area of adaxial epidermal cells. (F) Total cells per leaf calculated from (d) and (e). (g) Representative leaves from WT-717 and three *BL-SRDX*-oe lines. (h) Validation of the over-expression of *BL*-SRDX in the three lines (1, 3, and 8). Error bars represent SE (n = 5 leaves from the three lines, in e n = 20 are the cells from leaves from of the three lines). Asterisks indicate significance as determined by Student’s t-test, with * denoting P <0.05.
(TIF)

S6 Fig Stem development in BL transgenics. Stem cross sections from different genotypes are shown as follows: (a), (d), and (g) WT-717; (b), (e), and (h) *BL-oe*; and (c), (f), and (i) *BL*-i. All stem sections were stained with toluidine blue and observed under phase contrast at different internodes as follows: (a) to (c) 5^th^ internode; (d) to (f) 10^th^ internode; and (g) to (i) 20^th^ internode. Note reduced lignified (not as bright) in *BL-oe* 5^th^ internode (b). (j) Stem diameter. (k) Xylem width. (l) Internode number. (m) Plant height. Error bars in (j) to (m) are SE (n = 5). Pf—phloem fibers, Xy—xylem, Pi—pith. Scale bar = 500 μm.
(TIF)

S7 Fig Expression of *AINTEGUMENTA* in young leaves of *BL* transgenics. RT-qPCR Relative expression was normalized using ubiquitin (UBI) (n = 3, mean ±SE). Asterisks indicate significance as determined by Student’s t-test, with * denoting P <0.05.
(TIF)

S8 Fig Validation of microarray results. For comparison, RT-PCR and microarrays expression are shown side by side. Bars represent mean ±SE (n = 3 for PCR, n = 2 for microarray). Abbreviations used correspond to the names and gene models specified in S2 Table. Quantitative RT-PCR expression estimates were normalized using ubiquitin.
(TIF)

S1 Data Deferentially expressed genes in BL-oe. (XLSX)

S2 Data Common deferentially expressed genes in apex, leaf, and stem. (XLSX)

S3 Data Gene ontology enrichment among deferentially expressed genes analyzed by tissues' specific DEG. (XLSX)

S4 Data Comperison of the GO enrichment among deferent tissues' specific DEG. (XLSX)

S5 Data Subset of DEG genes involved in plant growth [51, 52]. (XLSX)

S6 Data Subset of DEG genes related to root system development (GO:0022622). (XLSX)
