# Supplementary material for: BIG LEAF is a regulator of organ size and adventitious root formation in poplar
Source: PLoS One. 2017 Jul 7;12(7):e0180527. doi: 10.1371/journal.pone.0180527 (PMC5501567; doi:10.1371/journal.pone.0180527)
Supplement: S1 Table — (PDF) [file pone.0180527.s001.pdf]

S1 Table. Primers used in this work

Primer name	Purpose	Sequence 5'>3'
p2735CI645F	Transgene verification	CTCAGAATGTCTTCCTCCTCCTCCTCCTCA
p2735CI645R	Transgene verification	TCTAGACTACTGTGCCCCAAAATC
NPTf	Transgene verification	ATCAGGATGATCTGGACGAAGAG
NPTr	Transgene verification	GATACCGTAAAGCACGAGGAAG
GFPf	Transgene verification	TGGCGATGGCCCTGTCCTTT
GFPr	Transgene verification	TGCCATGTGTAATCCCAGCAGC
GUS-L	Transgene verification, expression	CTGATAGCGCGTGACAAAAA
GUS-R	Transgene verification, expression	GGCACAGCACATCAAAGAGA
BL-B1	BL Cloning	GGGGACAAGTTTGTACAAAAAAGCAGGCTATGTCTTCCTCCTCCTCCTC
BL-B2	BL Cloning	GGGGACCACTTTGTACAAGAAAGCTGGGTCTGTGGGATTTTGGGGCACAGTAG
BL1L-B1	BL RNAi Cloning	GGGGACAAGTTTGTACAAAAAAGCAGGCTTCCTCCTCCTCACCATCATC
BL1R-B2	BL RNAi Cloning	GGGGACCACTTTGTACAAGAAAGCTGGGTGCAAGAGCTTCCAGAAAAGG
BLIntL-B1	BL RNAi Cloning	GGGGACAAGTTTGTACAAAAAAGCAGGCTAAGCTCTTGCTGCCCAAGTC
BLIntR-B2	BL RNAi Cloning	GGGGACCACTTTGTACAAGAAAGCTGGGTGGCGGTAGATATACTCCTCTCG
BL-R(-stop)B2	BL Cloning (no stop codon)	GGGGACCACTTTGTACAAGAAAGCTGGGTTCTGTGCCCCAAAATCCCACAG
BL-R(-stop)DN	BL Cloning (w/ SRDX fusion)	GGGGACCACTTTGTACAAGAAAGCTGGGTTCAAGCAAACCCTAAACGCAACTCCAAGTCTAAGTCAAGCTGTGCCCCAAAATCCCACAG
BLrt-F	Gene expression	TGTCCGCCTCTTTGATCTCACC
BLrt-R	Gene expression	ATGGTGTTCAGGGCGGAAAGTG
UBIf	Gene expression	AGAGTGTGAGAGAGAGAAGAG
UBIr	Gene expression	CGACGACCATCAAACAAGAAG
PDR9-F	Gene expression, Microarray validation	TCAGCAGAGCTTGGAGGAAGCA
PDR9-R	Gene expression, Microarray validation	AGCCAAGCATTGTGATGCGTCT
ATHB40-F	Gene expression, Microarray validation	ACGCCGATCTTAGTGGGGCA
ATHB40-R	Gene expression, Microarray validation	ACGGCAACTTGGCGAGGATCG
MYB62-F	Gene expression, Microarray validation	GTGCAGAAGGAGGCACGCCA
MYB62-R	Gene expression, Microarray validation	GGCGAGGAATTATGAACTGGGGGA
PtaANAC047-F	Gene expression, Microarray validation	TGGATGACTGGGTTCTTTGCCGGA
PtaANAC047-R	Gene expression, Microarray validation	GCTTGGTAAAGGGTTTTCTGGGACA
VRN1-F	Gene expression, Microarray validation	GCAGCAGCTGTCATCTCCAAACG
VRN1-R	Gene expression, Microarray validation	TGCTCTTTCTCGTTCCTCAGCAGT
DIN9-F	Gene expression, Microarray validation	TGCTCACATACAAACAGGGCTTCCC
DIN9-R	Gene expression, Microarray validation	TGCACATTGCACCGTCGCCA
CAX3-F	Gene expression, Microarray validation	ACGCACTGCTTCTGCTTTTG
CAX3-R	Gene expression, Microarray validation	TCCACTCCGGAAGCCTATCA
ARR9-F	Gene expression, Microarray validation	CTGCATGCCTGGAATGAGCGGT
ARR9-R	Gene expression, Microarray validation	CCTCTGCTCCTTCTTCCAAGCACA
DUF506-F	Gene expression, Microarray validation	GCTGGCGGATACAGCGAGGG
DUF506-R	Gene expression, Microarray validation	GGCGGGATAGGAGGGGGCTT
