# Supplementary material for: BIG LEAF is a regulator of organ size and adventitious root formation in poplar
Source: PLoS One. 2017 Jul 7;12(7):e0180527. doi: 10.1371/journal.pone.0180527 (PMC5501567; doi:10.1371/journal.pone.0180527)
Supplement: S2 Table — (PDF) [file pone.0180527.s002.pdf]

Table S2. Leaf thickness and chloroplast number/cell in *BL-oe*.

Genotype	Leaf thickness (µm)	Chloroplasts/cell (5 cells/section)
WT-717 Plant 1	93.4	7, 9, 11, 8, 9
WT-717 Plant 1	83.5	10, 7, 6, 9, 9
WT-717 Plant 1	79.6	8, 10, 7, 10, 8
WT-717 Plant 2	87.2	9, 10, 10, 10, 11
WT-717 Plant 2	92.8	10, 8, 9, 10, 6
WT-717 Plant 2	94.5	7, 6, 9, 6, 7
WT-717 Plant 3	120.3	6, 6, 10, 10, 6
WT-717 Plant 3	116.1	7, 9, 7, 6, 7
WT-717 Plant 3	97.3	12, 9, 6, 8, 10
WT-717 Plant 3	105.6	7, 9, 8, 6, 10
BL-oe line line 1	64.4	4, 3, 3, 5, 3
BL-oe line line 1	61.0	2, 3, 3, 1, 5
BL-oe line line 1	58.3	3, 3, 4, 5, 2
BL-oe line line 1	43.6	4, 4, 3, 3, 1
BL-oe line line 2	53.5	6, 1, 5, 5, 0
BL-oe line line 2	52.5	4, 5, 4, 5, 6
BL-oe line line 2	75.5	0, 4, 1, 0, 5
BL-oe line line 3	61.0	2, 2, 1, 3, 4
BL-oe line line 3	69.7	3, 6, 5, 2, 2
BL-oe line line 3	75.3	4, 1, 3, 3, 4

Data are from multiple leaves cross sections as shown in Fig 6. For the leaf thickness measurements the ImageJ (https://imagej.nih.gov/ij/) were used, and for chloroplast count the sections were 5µm thick.
