# Supplementary figures and images for: BIG LEAF is a regulator of organ size and adventitious root formation in poplar
Source: PLoS One. 2017 Jul 7;12(7):e0180527. doi: 10.1371/journal.pone.0180527 (PMC5501567; doi:10.1371/journal.pone.0180527)

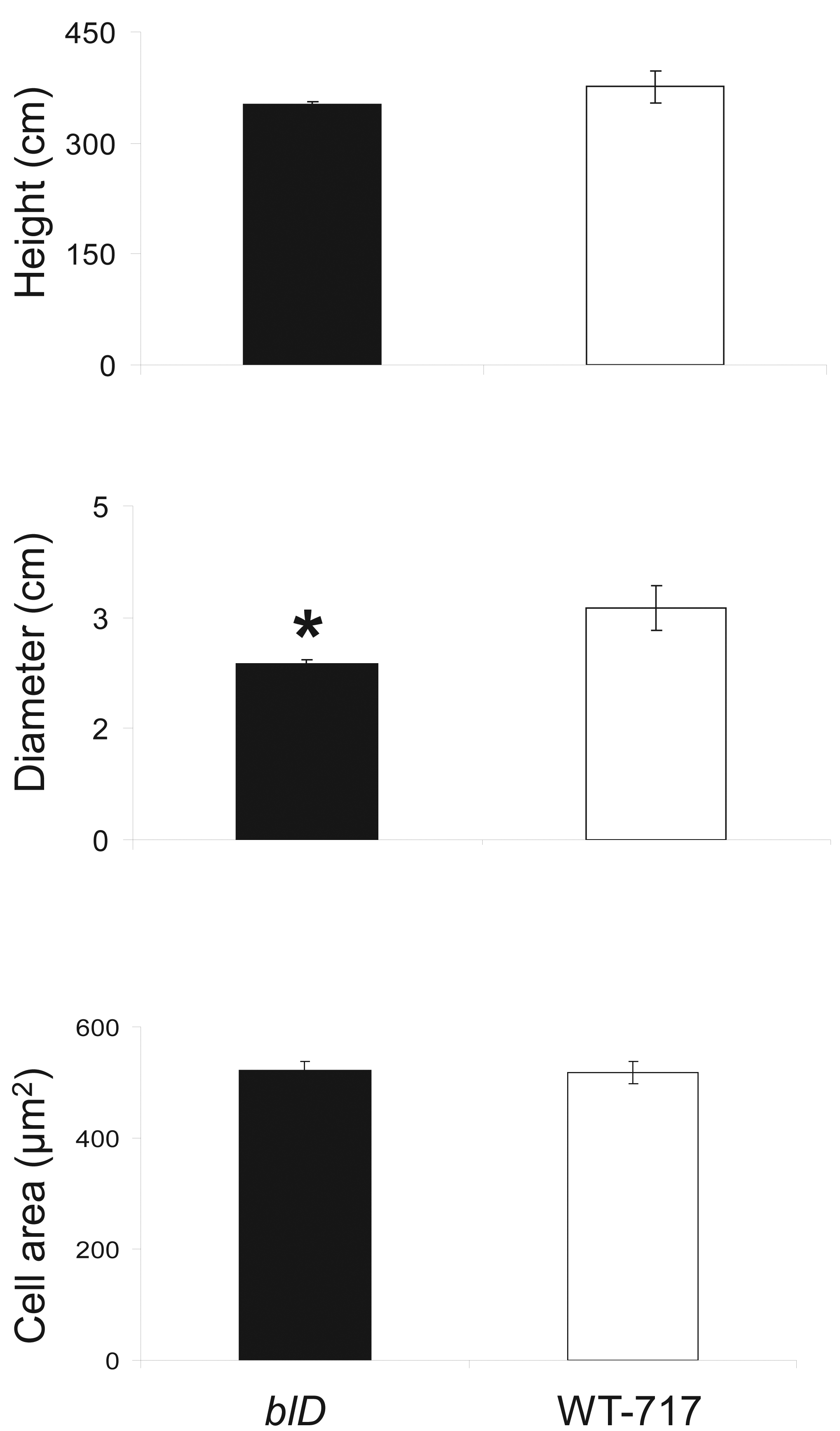

Supplement: S1 Fig — Graphs present data for the plants height (top), stem diameter (middle), and leaves adaxial epidermis cell area (bottom) are shown. Error bars represent mean ±SE (n = 4, n = 25 for cell area), asterisk indicate significance P<0.05 as determined by Student’s t-test. (TIF) [file pone.0180527.s003.tif]

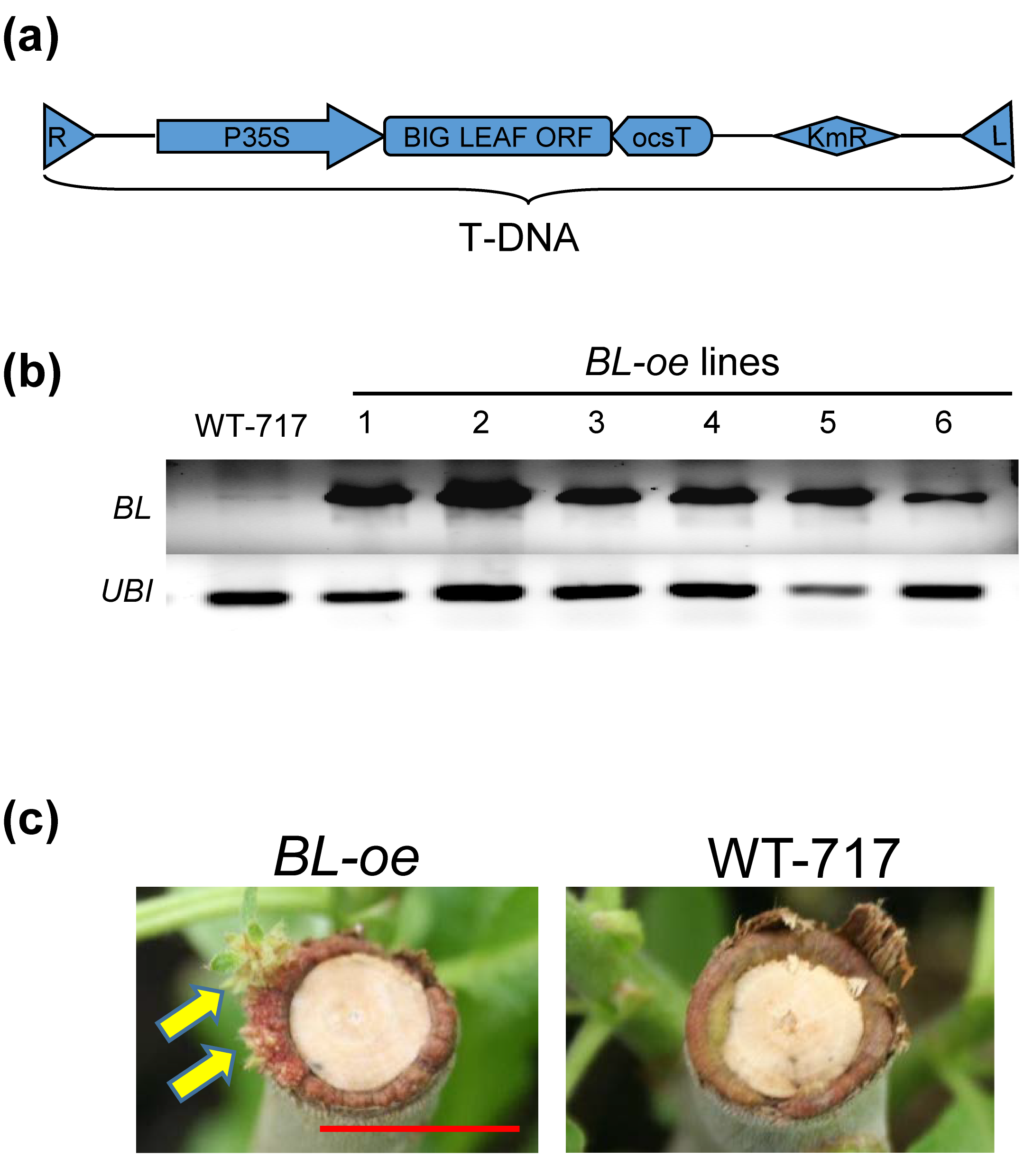

Supplement: S2 Fig — (a) Schematic representation of the construct used for transformation. Backbone plasmid is pART27. P35S = 35S promoter from the Cauliflower Mosaic Virus, ocsT = octopine synthase terminator, KmR = gene cassette for kanamycin resistance in plants, R and L are right- and left-hand T-DNA borders. (b) RT-PCR expression analyses of BL in apical shoots from six, randomly chosen transgenic BL-oe lines (1 to 6) reveal strong up-regulation of the gene in all tested lines. Ubiquitin (UBI) was used as a loading control. (c) Spontaneous shoot outgrowth from cambium-derived callus in BL-oe transgenic plants (left panel), observed in about 15% (with 1–4 shoots) of the plants. WT-717 (right panel) formed regular callus to seal the wound but no shoot outgrowth was observed. (TIF) [file pone.0180527.s004.tif]

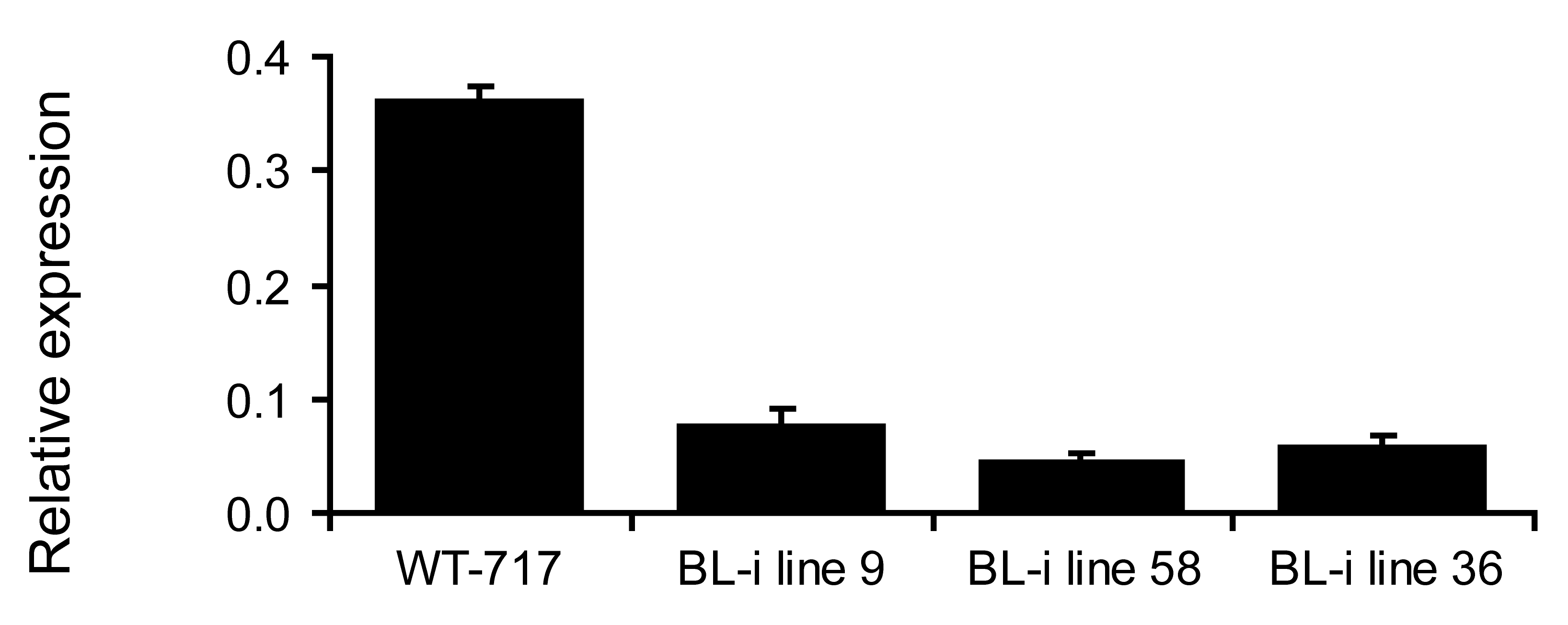

Supplement: S3 Fig — In upper panel is shown representative RT-PCR demonstrating down-regulation of BL expression in apex from three transgenic lines. Ubiquitin (UBI) is used as a loading control. (TIF) [file pone.0180527.s005.tif]

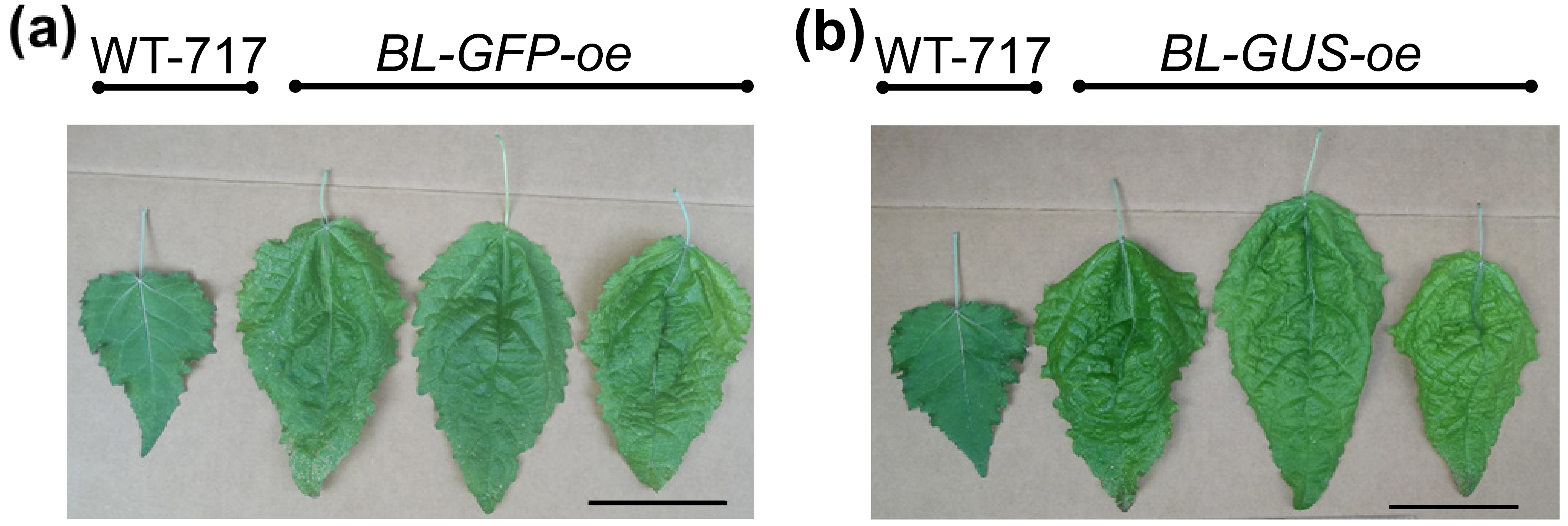

Supplement: S4 Fig — Over-expression of BL fused to either GFP (a) or GUS (b) fully recapitulated the BL-oe phenotype. Leaves shown are from three independent transgenic lines. Scale bar = 10 cm. (TIF) [file pone.0180527.s006.tif]

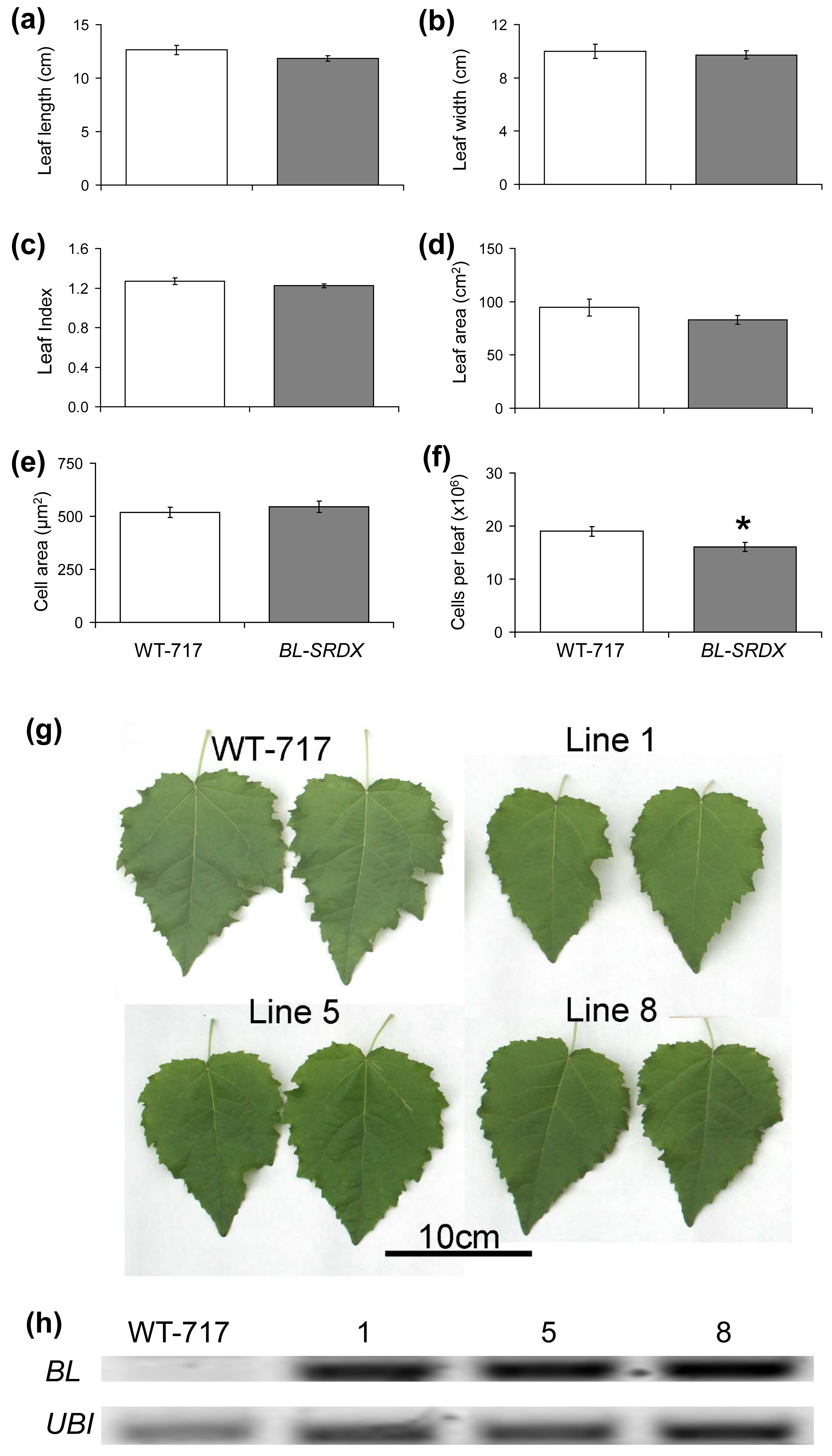

Supplement: S5 Fig — Leaf parameters were measured from 15th to 20th fully developed leaf. (a) Leaf length. (b) Leaf width measured at leaf center. (a) Leaf Index calculated from (a) and (b). (d) Leaf area. (e) Cell area of adaxial epidermal cells. (F) Total cells per leaf calculated from (d) and (e). (g) Representative leaves from WT-717 and three BL-SRDX-oe lines. (h) Validation of the over-expression of BL-SRDX in the three lines (1, 3, and 8). Error bars represent SE (n = 5 leaves from the three lines, in e n = 20 are the cells from leaves from of the three lines). Asterisks indicate significance as determined by Student’s t-test, with * denoting P <0.05. (TIF) [file pone.0180527.s007.tif]

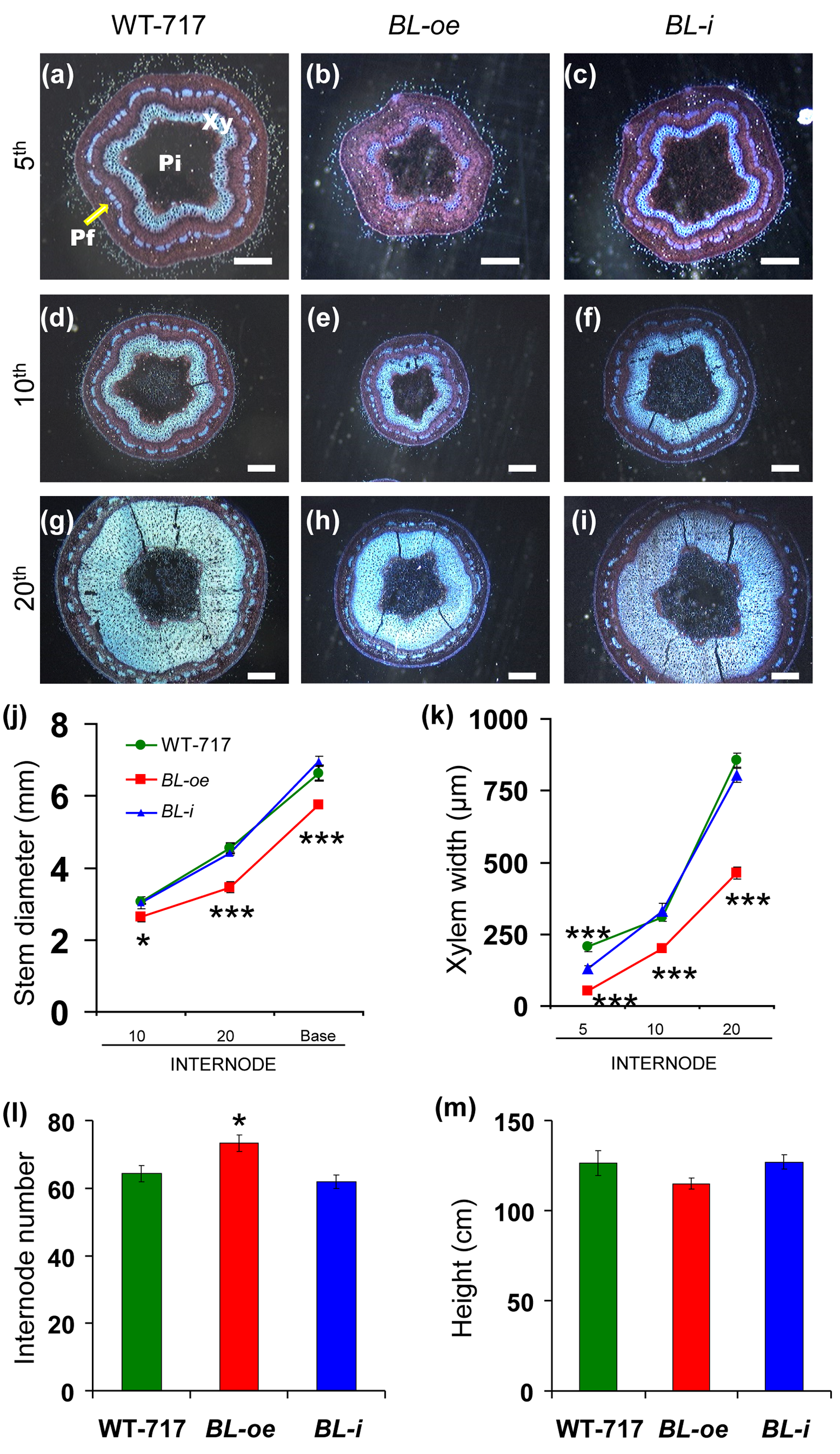

Supplement: S6 Fig — Stem cross sections from different genotypes are shown as follows: (a), (d), and (g) WT-717; (b), (e), and (h) BL-oe; and (c), (f), and (i) BL-i. All stem sections were stained with toluidine blue and observed under phase contrast at different internodes as follows: (a) to (c) 5th internode; (d) to (f) 10th internode; and (g) to (i) 20th internode. Note reduced lignified (not as bright) in BL-oe 5th internode (b). (j) Stem diameter. (k) Xylem width. (l) Internode number. (m) Plant height. Error bars in (j) to (m) are SE (n = 5). Pf—phloem fibers, Xy—xylem, Pi—pith. Scale bar = 500 μm. (TIF) [file pone.0180527.s008.tif]

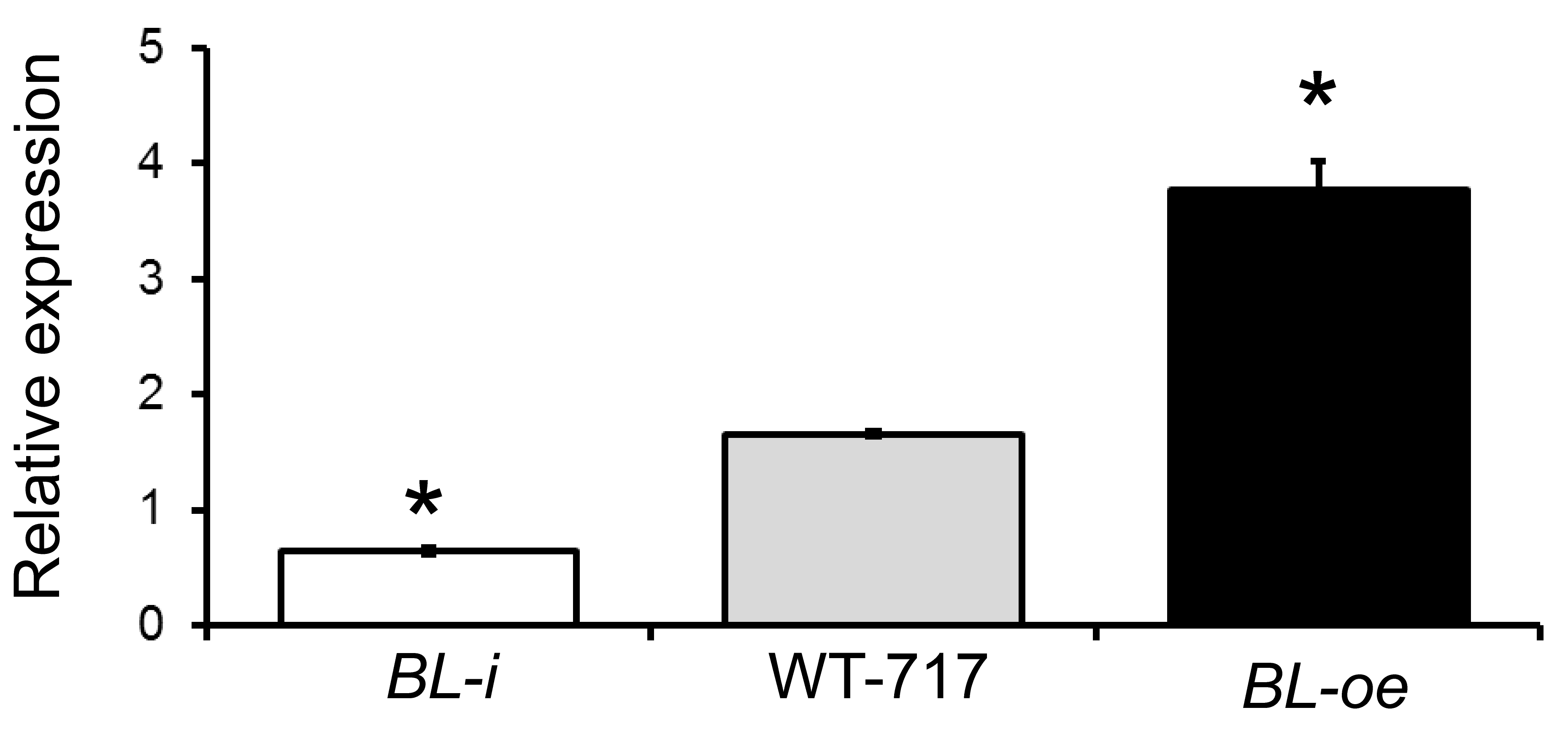

Supplement: S7 Fig — RT-qPCR Relative expression was normalized using ubiquitin (UBI) (n = 3, mean ±SE). Asterisks indicate significance as determined by Student’s t-test, with * denoting P <0.05. (TIF) [file pone.0180527.s009.tif]

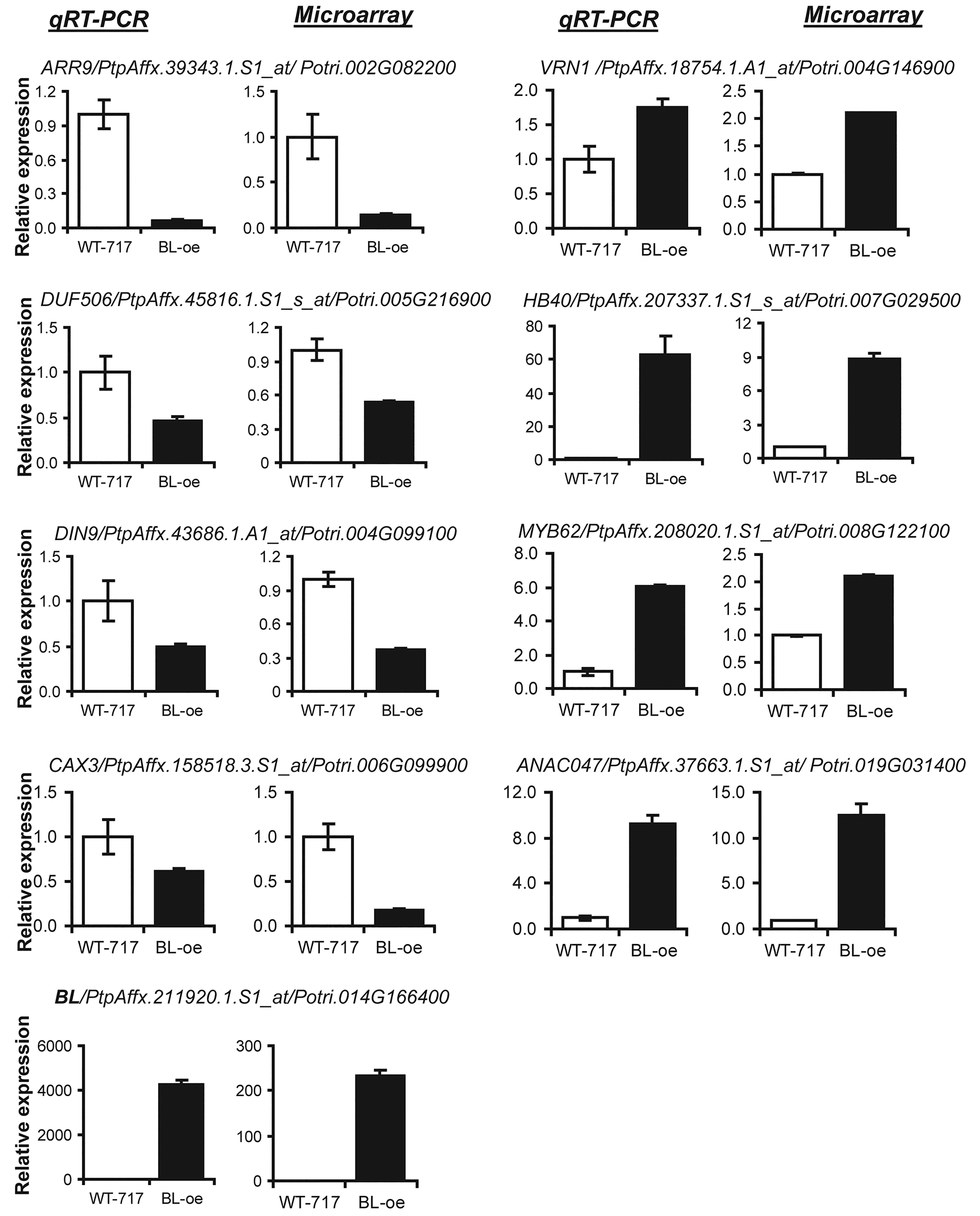

Supplement: S8 Fig — For comparison, RT-PCR and microarrays expression are shown side by side. Bars represent mean ±SE (n = 3 for PCR, n = 2 for microarray). Abbreviations used correspond to the names and gene models specified in S2 Table. Quantitative RT-PCR expression estimates were normalized using ubiquitin. (TIF) [file pone.0180527.s010.tif]
